# Influence of the Synthesis Method on the Textural and Morphological Characteristics of Ni-Based Mesoporous Molecular Sieves

**DOI:** 10.3390/ma18051012

**Published:** 2025-02-25

**Authors:** Dănuța Matei, Mihai Postelnicu, Sonia Mihai, Diana-Luciana Cursaru

**Affiliations:** Faculty of Petroleum Refining and Petrochemistry, Petroleum-Gas University of Ploiesti, 100680 Ploiesti, Romania; danuta.matei@upg-ploiesti.ro (D.M.); smihai@upg-ploiesti.ro (S.M.)

**Keywords:** MCM-41, MCM-48, SBA-15, mesoporous materials, incorporation, impregnation, Ni-MCM-41

## Abstract

Purely siliceous MCM-41, MCM-48, SBA-15, and Ni-containing molecular sieves were synthesized by the sol–gel method. The impact of the Ni loaded by incorporation and impregnation in the framework of molecular sieves on the textural and morphological characteristics of the solids was comprehensively investigated. The incorporation method proved to be more effective in terms of textural and morphological properties; therefore, we also investigated the influence of Ni incorporation on the structure of MCM-41 at different loadings (3, 6 and 9 wt.%). Moreover, all solids were characterized by FT-IR, TGA, XRD, SEM-EDS, and N_2_ adsorption. The resulting mesoporous materials exhibit a porous structure with well-defined pore sizes of about 2.0–5.0 nm and high specific surface areas (634 m^2^g^−1^ for SBA-15, 1592 m^2^g^−1^ for MCM-48, and 1769 m^2^g^−1^ for MCM-41) alongside uniform pore size distributions. The MCM-41 structure remained unchanged after loading of Ni; however, its surface area and pore diameter decreased due to pore blockage.

## 1. Introduction

Mesoporous materials are very versatile and therefore are utilized in a multitude of applications, including adsorption, separation, or as catalysts. The most utilized ordered mesoporous materials are those based on the MCM-41, MCM-48, and SBA-15 structures.

MCM-41 (Mobile Crystalline Material) are silicates obtained by hydrothermal synthesis with a liquid templating mechanism and have pores with a well-defined size and uniform shape with hexagon channels; a high surface area of 1000 m^2^/g; excellent thermal, hydrothermal, and hydrolytic stability; and high porosity [1]. MCM-41, along with MCM-48, are included in the M41S family of silicate/aluminosilicate mesoporous molecular sieves discovered by Mobil Corporation in 1992 and the have different mesophases: hexagonal for MCM-41 and cubic for MCM-48 [1,2,3,4,5,6,7,8,9,10]. SBA-15 (Santa Barbara Amorphous) is a mesoporous material characterized by its hexagonal pore arrangement and was first produced in 1998. It has a larger pore size than MCM-41 and MCM-48, with better thermal, mechanical, and chemical resistance properties, being a preferable choice for catalyst use [1,2,6,9,11,12].

Usually, the synthesis of a mesoporous material consists of the dissolution of template molecules in the solvent, which is then followed by the addition of silica sources like tetraethyl-orthosilicate (TEOS), metasilicate, etc. [5,6]. This synthesis involves: a solvent like water and/or ethanol, a silica precursor like TEOS/TBOS (tetrabuthyl-orthosilicate), a surfactant, and a catalyst [5]. For the next step, the solution is stirred at a certain temperature to allow hydrolysis and condensation. To control the condensation process, the temperature is increased after a specific period. Ultimately, the products will be recovered, washed, dried, and calcinated for templated removal. The templated removal could also use some extraction methods [5,6]. The synthesis method discussed here is the hydrothermal method, which is the most widely used technique. However, other methods have also been studied, including microwave treatment of the precursor gel. This alternative approach results in a reduced reaction time and improved control over the texture and morphology [5,13,14].

To achieve the template, three methods could be applied: an ionic surfactant, a neutral surfactant, or no surfactant [5]. Using an ionic surfactant to form the template usually involves a mechanism of liquid crystal templating, where the central structure is an organic species surrounded by inorganic oxides that form the framework [5]. As Bhattacharyya and collaborators described in their review, the long-chain surfactant molecules are assisted by micelle self-assembly to form a liquid–crystalline phase. The silicate species deposits between surfactant rods condense and form an inorganic network, exhibiting hexagonal ordering influenced by the interaction between the surfactant and silicate species. After surfactant template removal, the pore size is between 2 and 10 nm [5]. The neutral surfactant templated method employs a hydrogen-bonding pathway to create cubic mesoporous structures. The silica species generated by the hydrolysis of silica precursors interact with neutral surfactants by hydrogen bonding. These hydrogen bonds will change the volume ratio of head to chain and will facilitate the assembly of rod-like micelles. In the end, further hydrolysis and condensation of the silica precursor takes place, which will improve micelles formation and create the framework walls [5]. In the non-surfactant templated method, organic compounds such as glucose, urea, and maltose are utilized as templates for the pore structure in sol–gel processes. After template removal by solvent extraction, the surface area of the material is around 1000 m^2^/g and the pore diameter is between 2 and 6 nm. By varying the non-surfactant content in the solution, the pore diameter can be controlled [5].

The key parameters in the synthesis of MCM-41 and MCM-48 are alkalinity, synthesis time, and temperature [6].

One significant way to modify the properties of mesoporous materials is by incorporating organic and inorganic components [1]. This incorporation could take place on a silicate surface, inside the channels, or on the walls. Organic groups in mesoporous materials modify surface properties such as hydrophilicity, acidity, basicity, and hydrophobicity. This modification protects the surface from chemical attacks and enhances the binding of guest molecules [1].

By introducing various transitional metals into molecular sieves, they are modified and can either act as potential catalysts for hydrodearomatization and desulfurization of fuels or increase their adsorption capacity for photodegradation applications [4,15,16,17].

The utilization of Ni-SBA-15 and Ni-MCM-41 as adsorbents for hydrogen was studied by Carraro and collaborators [18,19]. Good results were obtained for a Ni-SBA-15 with 2.1 wt.% loading and Ni-MCM-41 with 2.5 wt.% loading, having a maximum hydrogen adsorption capacity at −196 °C [18,19]. Yuehong Shu and collaborators studied the adsorption of methyl blue on Ni-MCM-41 from aqueous solution [4]. It was found that a higher adsorption capacity is achieved with Ni-MCM-41 with 1 wt.% loading than for MCM-41 [4]. Borcănescu et al. studied the adsorption of CO_2_ on amino-functionalized molecular sieves of MCM-48 [20]. The mesoporous silica MCM-48 was functionalized with 3-glycidyloxypropyl trimethoxysilane (KH560) and then two amination reagents like ethylene diamine (N2) and diethylene triamine (N3) were used. Good results in CO_2_ adsorption capacities were observed for MCM-48 sil KH560-N3 at 30 °C [20]. The MCM-41 material has comparable adsorption ability for C_8_–C_12_ similar to commercial carbon adsorbents. Also, a full range of C_4_–C_12_ volatile organic compounds could be efficiently trapped by MCM-48 with a pore size of 3.7 nm at −20 °C [21].

Xinbin Yu and Christopher T. Williams describe several applications in [22] that involved the use of the Ni-SBA-16 catalyst for CO_2_ methanation, producing a CO_2_ conversion of 31% and a CH_4_ selectivity of 33% compared to 21.7% Ni-SBA-16.

Dry reforming of methane over Ni-SBA-15 is another example that resulted in a CO_2_ conversion of 64.2% and a CH_4_ conversion of 53.7% with a 23 wt.% Ni-SBA-15 catalyst [23]. A conversion rate of 95% and selectivity of 74% for n-C8 has been achieved with the hydrodeoxygenation of octanoic acid on bimetallic mesoporous silica modified with Ni, as another example of a Ni-modified mesoporous silica [23]. Carraro et al. investigated the use of Ni-SBA-15 (2.1 wt.%Ni) and Ni-MCM-41 (2.5 wt.%Ni) catalysts to absorb hydrogen and found that they could achieve the maximum amount with both catalysts [18,19].

Yuehong Shu et al. studied the adsorption of methyl blue on a Ni-MCM-41 (1 wt.% Ni) catalyst in aqueous solution [4]. Nickel-modified mesoporous silica has been studied extensively in the literature, particularly for ethylene oligomerization. Elsa Koninckx et al. showed in their paper that for different Si/Al ratios at the same Ni loading (0.5 wt.%) for Ni-MCM-41 and Ni-MCM-48 catalysts used in ethylene oligomerization the main product that is obtained is butene (41–49 mol %) and hexene (15–37 mol %), respectively [24]. Olivier-Bourbigou et al. in their review paper about nickel-catalyzed olefin oligomerization and dimerization [6] showed that most of the studies on the mesoporous silica-supported nickel catalysts were performed for ethylene oligomerization over aluminated MCM-41 and SBA-15 catalysts at different Ni loads of 0.5, 1, 2.6, and 5 wt.%. Propylene oligomerization studies on mesoporous silica catalysts like MCM-41, MCM-48, and SBA-15 are not as prevalent, and are mostly conducted on ZSM catalysts [25].

This study examined the effects of different synthesis methods on the textural and morphological properties of molecular sieves MCM-41, MCM-48, and SBA-15, as well as their variants loaded with 3 wt.% Ni. By understanding these relationships, we can enhance the design and application of these materials. The synthesis method employed was the sol–gel technique, while the incorporation (inc) and impregnation (imp) methods were used to load the metal into the molecular sieve framework. Various characterization techniques such as Fourier transformation infrared spectroscopy (FTIR), X-ray diffraction (XRD), scanning electron microscopy (SEM) coupled with energy-dispersive X-ray spectroscopy (EDS), and N_2_ adsorption and thermogravimetric analysis (TGA) were applied to analyze the changes in the physicochemical properties of molecular sieves due to the loading of Ni. These techniques also evaluated the effects of different concentrations of Ni incorporated into the MCM-41 framework and provided insights into the adsorption characteristics or their potential as catalysts. Our next investigation, based on the textural and morphological features of the Ni-based molecular sieves, aims to select the most suitable catalysts for propylene oligomerization to obtain fuels.

## 2. Materials and Methods

### 2.1. Materials and Reagents

The materials used for the synthesis of MCM-41 and MCM-48 molecular sieves included a silica source such as tetraethyl-orthosilicate (TEOS 99%), a structural agent such as cetyltrimethylammonium bromide (CTAB 99%), absolute ethanol (99.5%), and NH_4_OH (29% p/p), which were purchased from Sigma-Aldrich Chemical Co. (St. Louis, MO, USA), and deionized water. All the reagents used for the synthesis of SBA-15, including tetraethyl orthosilicate (99%), PEG-PPG-PEG (Pluronic P123; Mw~5800), and hydrochloric acid (HCl 35%) were purchased from Sigma-Aldrich Chemical Co.

Nickel sulfate NiSO_4_∙6H_2_O was purchased from Carl Roth GmbH (Karlsruhe, Germany).

### 2.2. Synthesis of Molecular Sieves

#### 2.2.1. Synthesis of MCM-41

Purely siliceous MCM-41 was synthesized according to the procedure described previously [25]. Briefly, a solution of 1.5 g of cetyltrimethylammonium bromide (C_19_H_42_BrN-CTAB) and 288 cm^3^ deionized water was prepared. After adding 102 cm^3^ of ethanol to this mixture, it was stirred for 1 h at 900 rpm. Then, 30 cm^3^ of ammonium solution (NH_4_OH) (29%) was added, and after solubilization, 6 cm^3^ of tetraethyl orthosilicate SiC_8_H_20_O_4_ (TEOS) was incorporated. The resulting suspension was stirred at 700 rpm for 3 h at room temperature. Afterward, the solid part was recovered by filtration with a vacuum pump and dried overnight in an oven at 60 °C. The sample was then calcined in an air current at 550 °C for 5 h, with a ramp of 5 °C/min.

#### 2.2.2. Synthesis of MCM-48

The direct sol–gel method was used to synthesize MCM-48 following the procedure that was previously described [26]. The first step was to dissolve 2.54 g CTAB in a solution of ethanol and 50 cm^3^ deionized water while stirring at 500 rpm at room temperature until a clear solution was obtained. After that, 15 cm^3^ of ammonia solution was incorporated into this solution. After 10 min, 3.64 cm^3^ of TEOS was slowly added to the solution mixture. The mixed solution was stirred continuously at 700 rpm for 2 h. The precipitate was collected by filtration with a vacuum pump and washed with deionized water until the solution became neutral. The sample was dried in an oven at 100 °C overnight and calcined at 550 °C for 5 h in the air flow, with a ramp of 5 °C/min.

#### 2.2.3. Synthesis of SBA-15

SBA-15 was synthesized by a hydrothermal method according to the reported method [27,28]. First, 6 g of triblock pluronic P123 (EO_20_-PO_70_EO_20_) was dissolved in 195 cm^3^ deionized water. Over the mixture was added 30 g of HCl (35%) stirring continuously for 6 h at 35 °C. Then, 12.5 g of TEOS was added to the solution and stirred for 5 min. The solution was then aged for 24 h at 35 °C, followed by hydrothermal treatment in an oven at 100 °C for 24 h. Vacuum filtration was used to separate the white solid product, which was dried overnight at 80 °C and finally calcined at 550 °C for 5 h in the air (with a ramp of 5 °C/min).

### 2.3. Metal (Ni)-Molecular Sieve Synthesis

NiSO_4_∙6H_2_O served as the Ni precursor to synthesize Ni-MCM-41, Ni-MCM-48 and Ni-SBA-15. A solution was made by dissolving 6 g of salt in 100 cm^3^ of distilled water.

#### 2.3.1. Synthesis of Ni-Molecular Sieves by Incorporation

Ni was incorporated into the framework of molecular sieves during the synthesis methods of MCM-41, MCM-48, and SBA-15 by introducing the silica source (TEOS), having the following molar composition ratios 1Si[TEOS]: *x*NiSO_4_∙6H_2_O, where *x* = 1.76 (3 wt.% Ni); 3.52 (6 wt.% Ni) and 5.3 (9 wt.% Ni). The pH of the MCM-41 and MCM-48 samples was adjusted to 12 by using ammonium solution before calcination at 550 °C for 5 h in the airflow. The pH of the SBA-15 samples was maintained at 2 because of the synthesis environment and then rebalanced with HCl if necessary.

#### 2.3.2. Synthesis of Ni-Molecular Sieves by Impregnation

First, 1.34 g of NiSO_4_∙6H_2_O was dissolved in different HCl volumes depending on the molecular sieve’s adsorption capacity, namely 19 cm^3^ for MCM-41 and MCM-48 and 17 cm^3^ for SBA-15. Next, 10 g of the molecular sieve was dipped in this solution. The pH of the solution was adjusted to 12 using an ammonium solution for the Ni-based MCM-41 and MCM-48 samples, while the pH of the Ni-based SBA-15 samples was kept at 2. The mixture was stirred vigorously for 1 h and then placed in an oven at 60 °C for 48 h. Afterward, the sample was ground and calcined at 550 °C for 5 h in an air current.

## 3. Results and Discussion

### 3.1. Sample Characterization

The structures of all prepared samples were analyzed by X-ray diffraction of the powder to obtain information about their crystallographic phase, structure, and chemical composition. The XRD spectra were obtained using a Bruker D8 Advance diffractometer (Karlsruhe, Germany; type θ-θ). Parameters were Cu-K radiation (λ = 1.5418 nm), 40 kV, and 40 mA.

The materials were investigated by Fourier transform infrared spectroscopy (FT-IR). The FT-IR spectra of the synthesized samples were detected using an FT-IR Shimadzu IRTracer-100 spectrophotometer (Kyoto, Japan) with a scanning range of 400–4000 cm^−1^.

The specific surface and textural properties of all samples were investigated using N_2_ adsorption–desorption isotherms to 77 K by using Quantachrome Nova 2200e equipment (BET area, pore volume, and pore size distribution; Quantachrome Tools, Boynton Beach, FL, USA). The samples were outgassed in a vacuum at 150 °C for 3 h before the sorption analysis. Properties were calculated using NovaWin software 1.0 (Boca Raton, FL, USA). The specific area was determined by the Brunauer–Emmett–Teller method (BET) in a pressure range of 0.05 ≤ p/p_0_ ≤ 0.3, while the pore size distributions were calculated with the adsorption BJH model.

The microstructural morphologies of the prepared samples were examined using an electronic scanning microscope (SEM, Scios 2 HIVAC Dual-Beam FIB-SEM with ultra-high resolution; Thermo Fisher, Brno, Czech Republic).

The use of thermogravimetric analysis (TGA) and differential thermogravimetric analysis (DTG) combined allowed for the measurement of thermal properties and synthesized materials. The measurements were made using a Setaram Labsys Evo S60/58986 TG analyzer (Cranbury, NJ, USA) using argon flow, increasing the temperature from 30 to 900 °C with a thermal gradient of 10 °C per minute.

### 3.2. X-Ray Diffraction (XRD)

**Figure 1:**
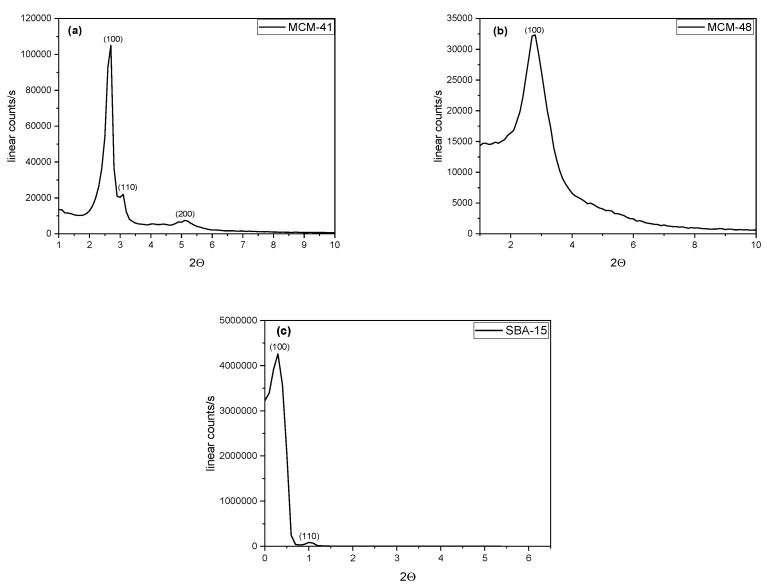
Low-angle XRD patterns of molecular sieves.

The crystalline phases and structural features of the molecular sieves expressed by the small angle XRD patterns are presented in Figure 1. MCM-41 (Figure 1a) exhibits three characteristic peaks: the first peak gives a sharp signal at 2θ = 2.6° due to (100) plane diffraction, and the other two peaks correspond to higher Miller index planes (110) and (200), indicating the formation of well-ordered mesoporous material [29,30]. MCM-48 presents a sharp (100) diffraction plane at 2θ = 2.8° (Figure 1b), while SBA-15 exhibits two characteristic peaks, a strong one at 2θ = 0.324° and a weak signal at 2θ = 1.045° corresponding to (100), (110) reflections (Figure 1c).

The crystallite size for all samples was also calculated. The Scherrer equation (1) gives a correspondence between the crystallite size (LC) and the full width of half maximum:

(1) $$LC=\frac{K\lambda }{\beta cos\theta }$$

where LC represents the mean size of the crystallites (nm), K is the shape factor equal to 0.94, λ is the wavelength of the X-rays (1.54 Å), θ is the Bragg angle (°), and β is the line broadening at half the maximum intensity peak (FWHM) in radians. Table 1 presents the crystallite sizes for the molecular sieves.

The average crystallite size of the molecular sieves ranged from 10.9 to 41.9, with the smallest crystallite size calculated for MCM-48.

**Figure 2:**
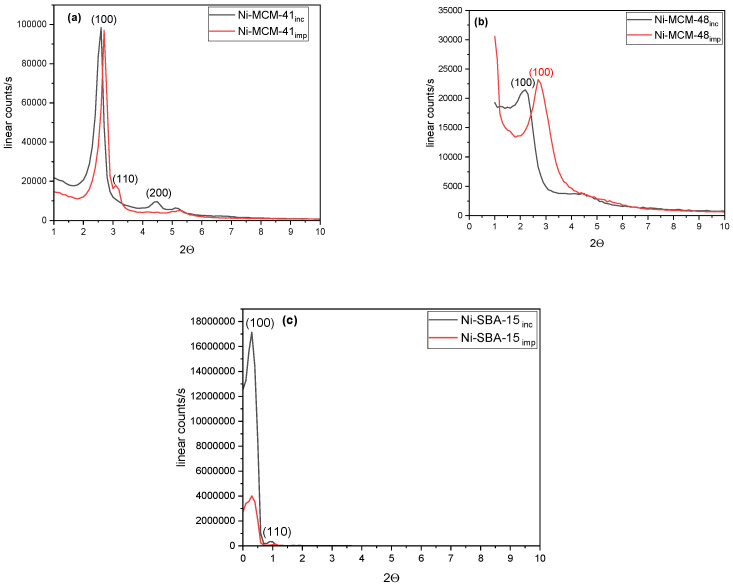
Low-angle XRD patterns of (**a**) Ni-MCM-41, (**b**) Ni-MCM-48, and (**c**) Ni-SBA-15 synthesized by incorporation (inc) and impregnation (imp).

This study investigated how the synthesis method affected the Ni-molecular sieve samples (Figure 2). Three diffraction peaks, indexed as (100), (110), and (200) reflections, are observed for the Ni-MCM-41 synthesized through incorporation and impregnation methods (Figure 2a). These peaks indicate a highly ordered mesoporous structure with a hexagonal pore array. Ni-SBA-15 samples (Figure 2c) had two distinctive peaks, which corresponded to (100) and (110). However, Ni-MCM-48 (Figure 2b) only displayed the distinctive peak of the diffraction plane (110). The presence of distinct peaks and their relative intensities reflect the structural ordering of the mesoporous materials [31,32]. Consequently, the reduction in intensity of the first peak and a noticeable broadening of all peaks can be attributed to a decrease in lattice order. The impregnation synthesis method results in a slight shift to the right of the peaks for the Ni-MCM-41 and Ni-MCM-48 samples. Additionally, the crystallite sizes of these samples are larger than those calculated for the samples synthesized through incorporation (Table 2).

**Figure 3:**
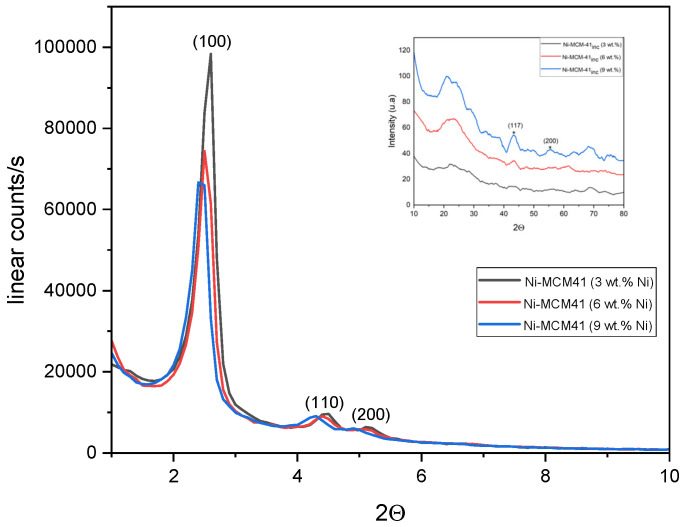
Low-angle XRD patterns for Ni-MCM-41 synthesized with different metal loadings and wide-angle XRD (* insets with (117) and (200) Miller indices).

By incorporating a larger quantity of metal into the MCM-14 framework (Figure 3), we observe the presence of the characteristic peaks corresponding to the Miller indices (100), (110), and (200) and the reflections are due to the ordered hexagonal array of parallel silica channels However, at a small angle, the characteristic diffraction peak intensities of the catalysts were weaker than those of MCM-41, and they decreased with increased metal loading. This indicates that the ordered structure of the MCM-41 has been damaged to a certain extent.

At a wide angle (the inset of the Figure 3), the diffractions showed obvious characteristic peaks of Ni particles (2θ = 44, 52 and 76°), while no obvious characteristic diffraction peaks of NiO particles (2θ = 37 and 43, 63°) could be observed, indicating the presence of the metallic Ni particles on the surfaces of the catalysts, which was also confirmed by SEM-EDS. The presence of NiO particles on the catalyst’s surface was impossible to determine. The particle size of Ni on the catalysts’ surface was calculated by Scherrer’s equation, and the results are shown in Table 3. It was also noticed that the size of the crystallites decreased as the metal loading increased.

### 3.3. FT-IR Investigations

**Figure 4:**
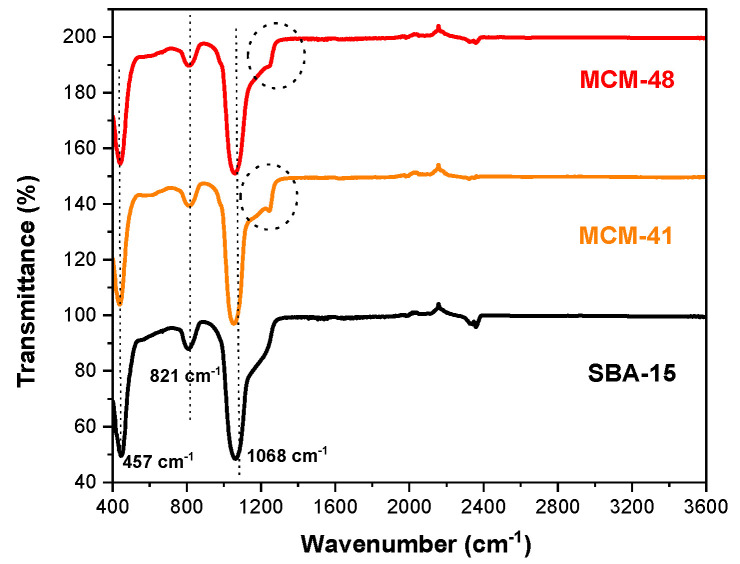
FT-IR spectra of MCM-48, MCM-41 and SBA-15.

**Figure 5:**
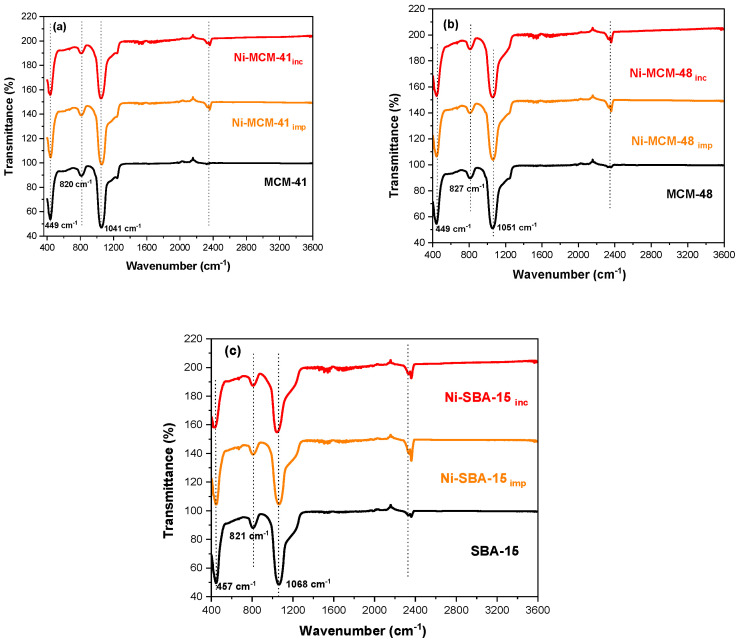
FT-IR spectra of (**a**) Ni-MCM-41, (**b**) Ni-MCM-48, and (**c**) Ni-SBA-15 synthesized by incorporation (inc) and impregnation (imp).

**Figure 6:**
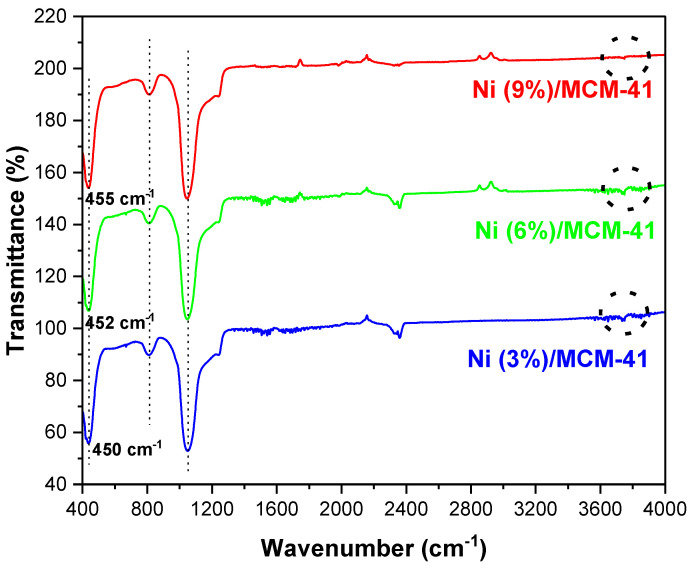
FT-IR spectra for Ni-MCM-41 synthesized with different Ni loadings.

The FT-IR spectra for the molecular sieves MCM-48, MCM-41, and SBA-15 were obtained in the wavenumber range of 3600 cm^−1^ to 400 cm^−1^ (Figure 4). These profiles include samples synthesized for Ni-MCM-14, Ni-MCM-48, and Ni-SBA-15 through incorporation and impregnation methods as shown in Figure 5. Additionally, Figure 6 presents the FT-IR spectra for Ni-MCM-41 with different nickel loadings of 3, 6, and 9 wt.% synthesized through incorporation.

The FT-IR spectra of mesoporous silica are relatively simple and well-assigned [33]. The Si-O vibration and Si-O bond stretching of surface Si-OH groups is shown at 457 cm^−1^. Also, the Si-O-Si stretching vibration of the SiO_4_ asymmetric band appeared at 1068 cm^−1^, while the symmetric one is at 821 cm^−1^. The silicate network is formed of Si-O-Si and many silanol Si-OH groups of different types [34]. At the same time, the shoulder at 1220 cm^−1^ which is more prominent in the cases of MCM-41 and MCM-48, is assigned to the Si-O-Si bridges, which are related to the transverse optic and longitudinal optic splitting mode [35].

This study investigated how the synthesis method affects Ni-molecular sieve samples (Figure 5). As expected, there were no significant changes in the FT-IR spectra following the incorporation or impregnation of nickel into the silica support. The main absorption bands of silica (~457 cm^−1^, ~821 cm^−1^, ~1068 cm^−1^) were preserved, indicating that the structure of the resulting samples was only slightly influenced. The FT-IR spectra of the Ni samples closely resemble those of the mesoporous supports. This indicates that the method used to impregnate or incorporate nickel into the mesoporous silica did not alter the texture of the material. Additionally, no bands associated with the Ni–O bond were detected within the studied range.

We can conclude that the coordinated metallic ions on the silica matrix cause only minor modifications to the catalyst’s spectrum compared to the spectrum of the silica support. This suggests that there was a low level of impregnation or incorporation of metal within the silica matrix, a fact that is also noted in the literature [36].

Although the Ni–O stretching vibration is overlapped by the dominant band at 449 cm^−1^ corresponding to the Si–O–Si or O–Si–O bending mode, the shifts from 449 cm^−1^ for the pure MCM-41 silica to 450 cm^−1^, 452 cm^−1^ and 456 cm^−1^ for Ni 3, 6 and 9 wt.%, respectively, can be observed in Figure 6. This also demonstrates the strong interactions between the Ni species and silica matrix [37].

### 3.4. N_2_ Adsorption–Desorption

**Figure 7:**
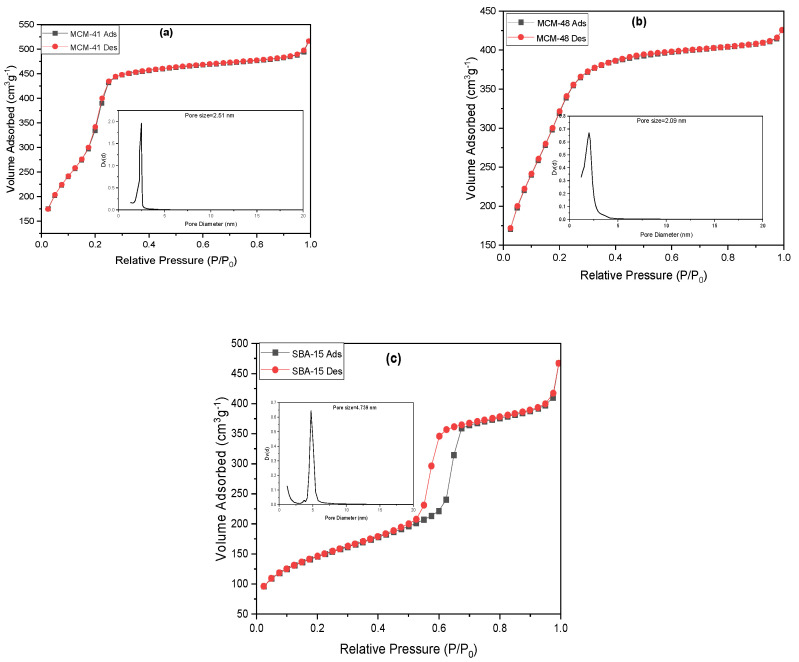
N_2_ adsorption–desorption isotherms for MCM-41 (**a**), MCM-48 (**b**) and SBA-15 (**c**) and BHJ pore size distribution curves (insets).

The nitrogen adsorption–desorption isotherms of calcined molecular sieves are shown in Figure 7. Based on the IUPAC classification, the N_2_ adsorption isotherms are classified as typical type IV [1]. Three distinct sections can be identified in the sorption isotherm for MCM-41 (Figure 7a) according to the pore-filling mechanism. Initially, at a relative pressure P/P_0_ ≈ 0.2, the adsorption occurs through the continuous film growth on the pore walls of MCM-41. Following this, within the pressure range 0.2 ≤ P/P_0_ ≤ 0.3, capillary condensation takes place within the core volume of the primary mesopores. Finally, the saturated vapor pressure is rapidly increasing due to the filling of the larger secondary mesopores with liquid N_2_. The isotherm is reversible with no hysteresis loop associated and the sample presents sharp capillary condensation, indicating a uniform structure.

The isotherm curve for MCM-48 (Figure 7b) complies with the usual Langmuir IV adsorption isotherms, with no hysteresis loop. The low relative pressures caused a significant inflection due to capillary condensation within mesopores. The uniformity of the pore distribution is reflected in the sharpness of the inflection. The pore size distribution patterns indicate a good agreement between these results and those observed. The adsorption capacity slows down as the relative pressure increases, resulting in adsorption on the outer surface of the mesoporous molecular sieve. The N_2_ adsorption–desorption isotherm for SBA-15 (Figure 7c) is classified as a type IV isotherm, with a hysteresis loop in the P/P_0_ range of 0.55 to 0.7, indicating capillary condensation within the pores of this mesoporous material.

Table 4 displays the textural parameters of the molecular sieves. The surface area decreases in the order S_MCM-41_ > S_MCM-48_ > S_SBA-15_. The same trend was observed for the pore volume, but the highest pore diameter was recorded for SBA-15 and the lowest for MCM-48.

**Figure 8:**
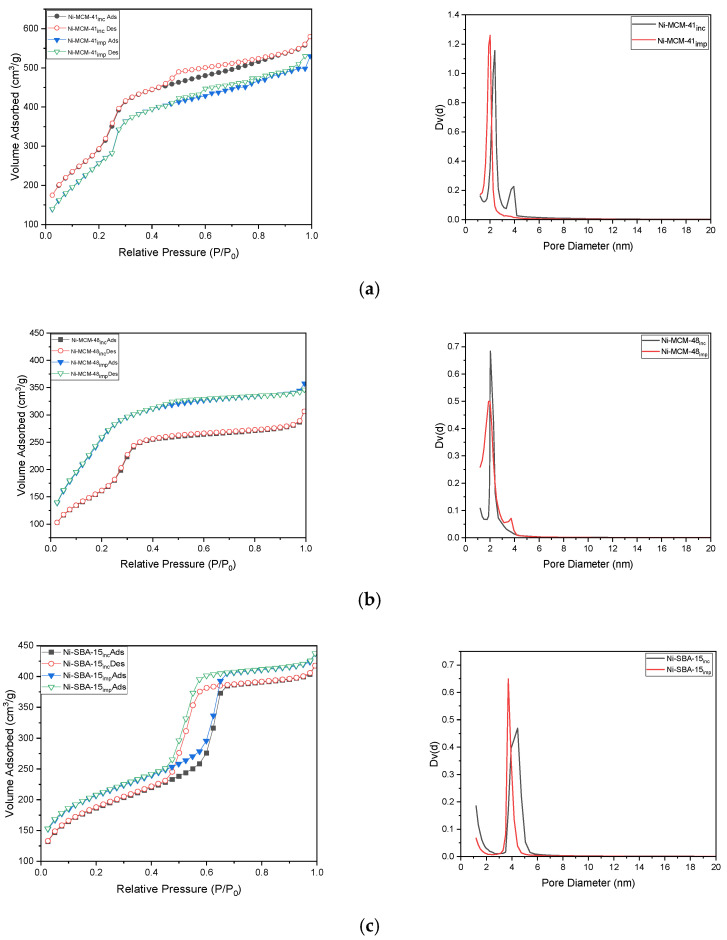
N_2_ adsorption–desorption isotherms (**a**) Ni-MCM-41, (**b**) Ni-MCM-48, and (**c**) Ni-SBA-15 synthesized by incorporation (inc) and impregnation (imp) and BHJ pore size distribution curves (insets).

By loading Ni into molecular sieves, it is observed that the desorption–adsorption isotherms follow the type of isotherms of molecular sieves, type IV for Ni-MCM-41 (Figure 8a) and Ni-MCM-48 (Figure 8b) and type V for Ni-SBA-15 (Figure 8c). The Ni-MCM-41 sample that was synthesized by incorporation also exhibits a hysteresis loop Type H1, which ranges from 0.4 to 0.8, which implies capillary condensation in the pores of this mesoporous material. This material is frequently linked to porous materials that have cylindrical-like pores [1].

It has also been observed that uniform mesopores can be obtained by using the synthesis method by incorporation.

The purely siliceous MCM-41 showed a high specific BET surface area of 1769 m^2^ g^−1^. However, when the Ni was incorporated into the MCM-41 structure, the specific BET surface area of the solids decreased at 1532 m^2^ g^−1^, while for Ni-MCM-41 synthesized by impregnation, the BET surface area decreased at 1512 m^2^ g^−1^. The total pore volume (0.81–1.12 cm^3^g^−1^) and the average pore diameter (2.0–2.4 nm) are consistent with the physical nature of the MCM-41 and Ni-MCM-41 mesoporous molecular sieves. In Table 5 are presented the textural parameters of the Ni-based molecular sieves samples synthesized by incorporation or impregnation.

The Ni-MCM-48 and Ni-SBA-15 samples showed a similar trend. MCM-48 was found to have a specific BET surface area of 1592 m^2^ g^−1^, which decreased when Ni is incorporated to 1271 m^2^ g^−1^ and impregnated to 792 m^2^ g^−1^. The specific BET area was SBA-15 > Ni-SBA-15_inc_ > Ni-SBA-15_imp_.

**Figure 9:**
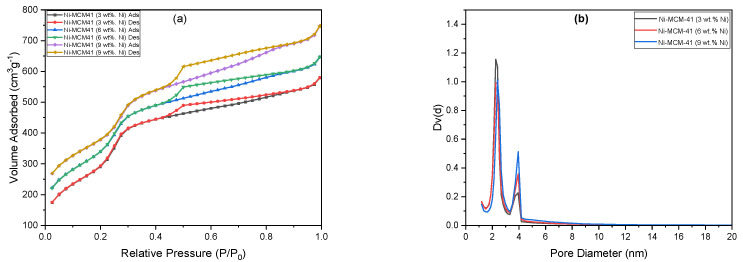
N_2_ adsorption–desorption isotherms (**a**) and BHJ pore size distribution curves (**b**) for Ni-MCM-41inc with different metal loadings.

Based on the pore-filling mechanism, the sorption isotherms for Ni-MCM-41 with different metal loadings can be divided into four distinct sections (Figure 9). At a relative pressure of P/P_0_ ≈ 0.2, the adsorption process begins with the continuous growth of a film on the pore walls of MCM-41. Following this, within the pressure range 0.2 ≤ P/P_0_ ≤ 0.3, the abruptness of the curve indicates a uniform size distribution of the pores. In the third section, capillary condensation occurs at a relative pressure range of 0.3 ≤ P/P_0_ ≤ 0.4, while, in the last section, the saturated vapor pressure is rapidly increasing due to the filling of the mesopores with liquid N_2_. The isotherms are reversible with an H1 hysteresis loop associated and the sample presents sharp capillary condensation, indicating a uniform structure of mesopores.

The surface area of Ni-MCM-41 (Table 6) consistently decreases as the content of nickel species increases. This reduction may be attributed to the formation of small nickel oxide nanoclusters, which block the pore channels of the samples. Furthermore, both the pore diameter and pore volume decline with higher nickel doping levels. This trend could be linked to the larger ionic radius of Ni^2+^ compared to that of Si^4+^, as well as the distinct differences in bond lengths and angles between the Si-O-Si and Si-O-Ni bonds. Consequently, nickel species are partially integrated into the hexagonal framework and walls of the silica network within MCM-41.

### 3.5. SEM-EDS Investigations

**Figure 10:**
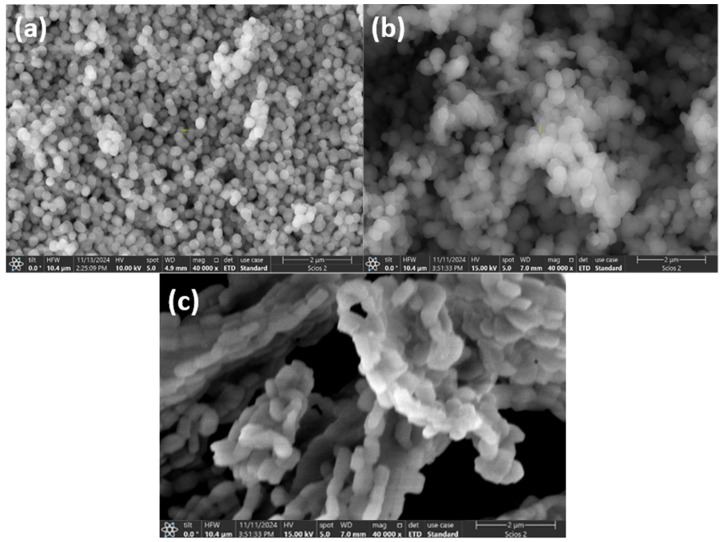
SEM images of (**a**) MCM-41, (**b**) MCM-48 and (**c**) SBA-15.

**Figure 11:**
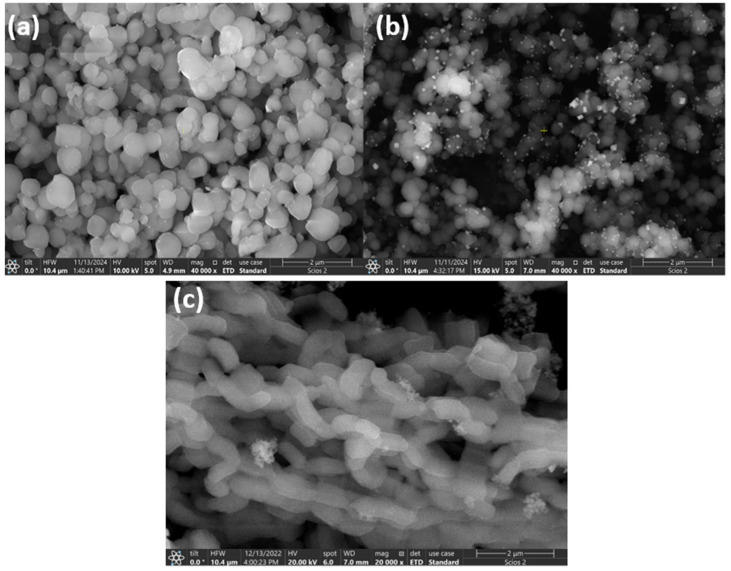
SEM images of Ni incorporated molecular sieves (**a**) Ni-MCM-41 (**b**) Ni-MCM-48 and (**c**) Ni-SBA-15.

**Figure 12:**
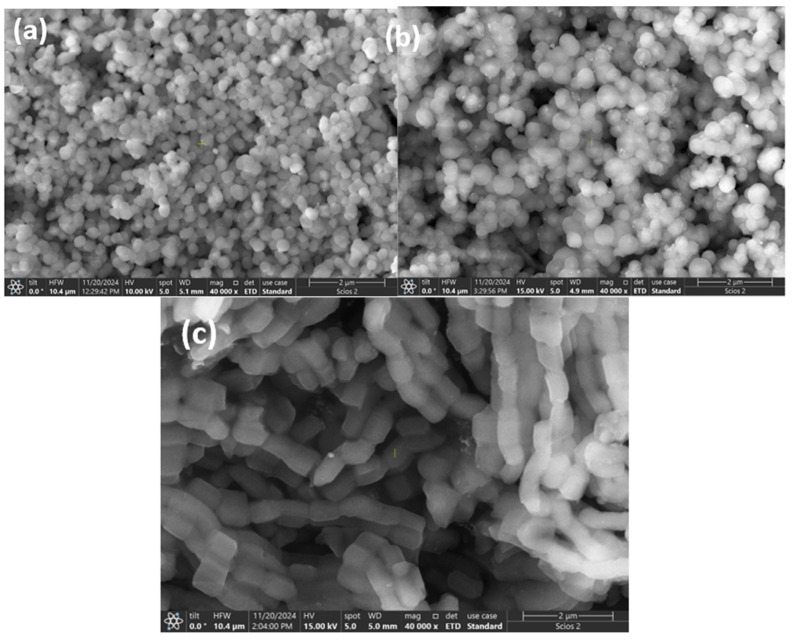
SEM images of Ni impregnated molecular sieves (**a**) Ni-MCM-41 (**b**) Ni-MCM-48 and (**c**) Ni-SBA-15.

**Figure 13:**
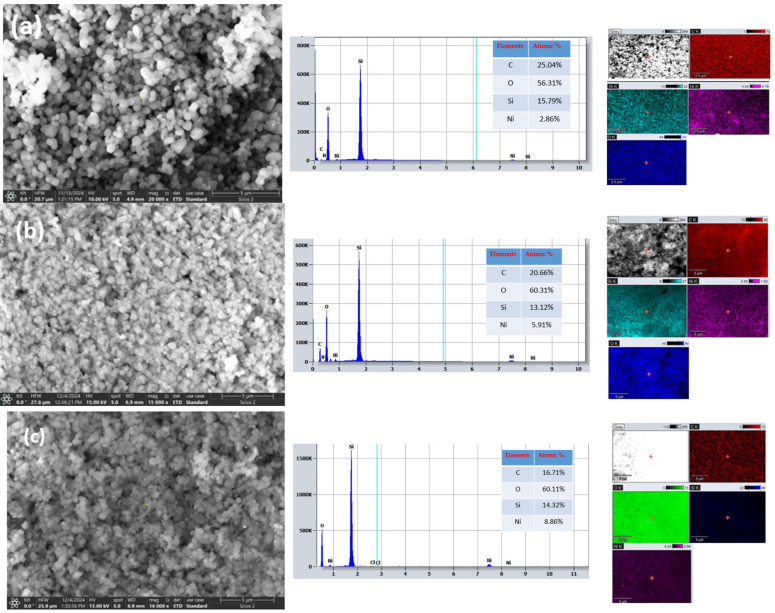
SEM images of the Ni-MCM-41 incorporated catalyst with (**a**) 3 wt.% Ni (**b**) 6 wt.% Ni, (**c**) 9 wt.% Ni with EDS spectra and elemental mappings of the samples.

The structure of the prepared mesoporous silicate nanoparticles, which is well-organized, was revealed by analyzing electron microscope images (Figure 10, Figure 11, Figure 12 and Figure 13) under the synthesis conditions previously presented.

From Figure 10a–c we can observe that the MCM-41 silica particles presented homogeneous spherical morphology and the MCM-48 particles showed a regular spherical morphology. The SBA-15 silica presents a rod-like morphology and the particles present aggregated macrostructures [38].

The SEM images of the silica supports and the Ni-molecular sieves exhibit a similar morphology, indicating that the mesoporous nature of the silica support is preserved even after metal impregnation or incorporation. The introduction of Ni ions into silica frameworks leads to a decrease in particle size due to weak electrostatic repulsion, which is characteristic of metal impregnation [25].

Direct synthesis by incorporation leads to better metal dispersion than material prepared by impregnation of pure silica, as shown.

The figure above illustrates that using mesoporous silica as a catalytic support results in the effective dispersion of metallic nanoparticles on its surface. The inserted element map image shows the presence and distribution of homogeneous Si, O, and Ni.

### 3.6. Thermogravimetric Analysis with Derivative Thermogravimetry (TGA-dTG)

**Figure 14:**
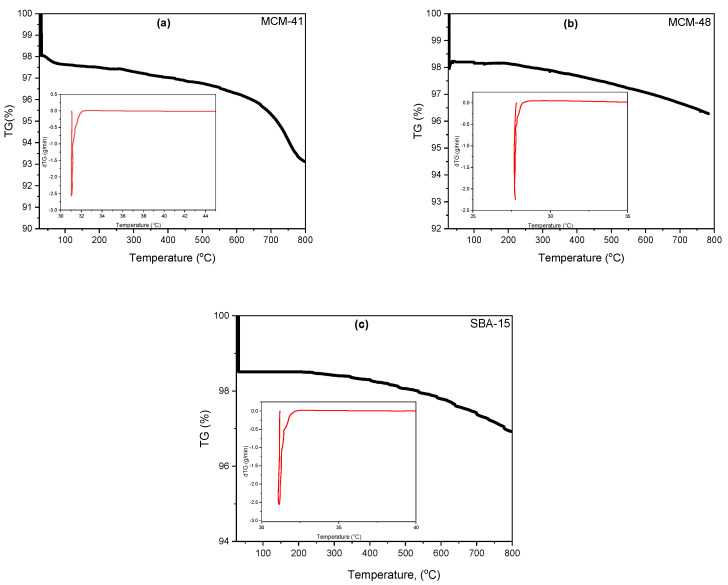
TGA and dTG (insets) thermograms of (**a**) MCM-41, (**b**) MCM-48 and (**c**) SBA-15.

**Figure 15:**
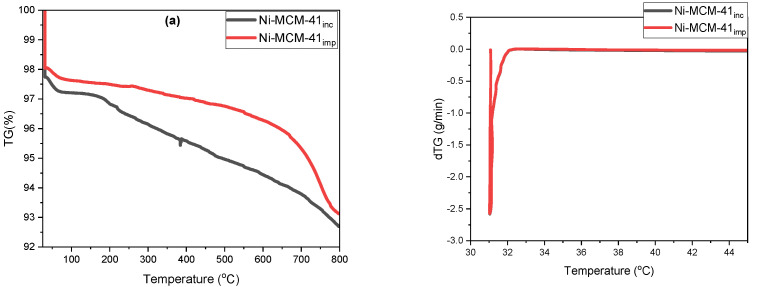

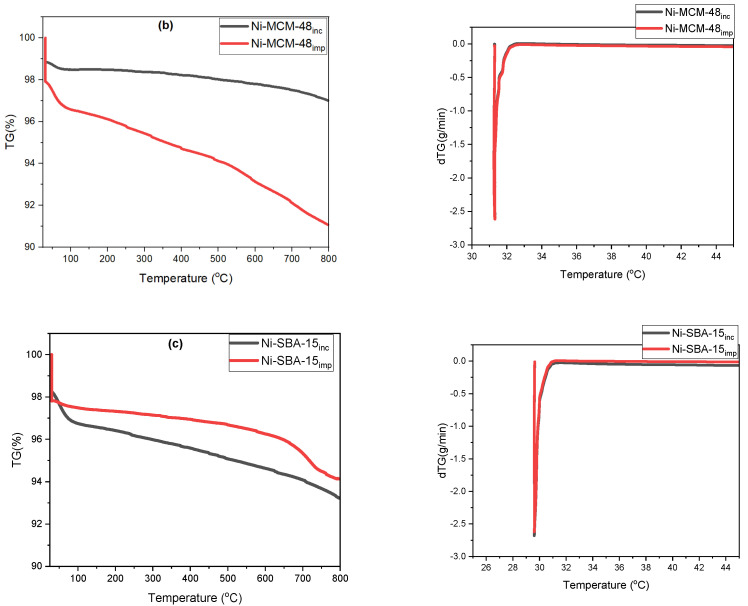
TGA (**left**) and dTG (**right**) thermograms of (**a**) Ni-MCM-41, (**b**) Ni-MCM-48 and (**c**) Ni-SBA-15.

**Figure 16:**
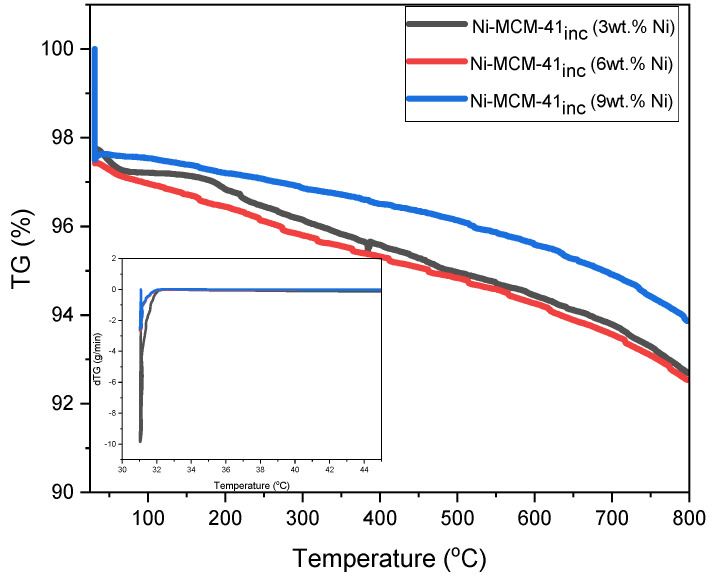
TGA and DTG (insets) thermograms of Ni-MCM-41 (3, 6, and 9 wt.% Ni).

The thermogravimetric analysis (TGA) and the corresponding differential thermogravimetric (dTG) profiles for the molecular sieves MCM-41, MCM-48, and SBA-15 were obtained from temperatures ranging from 30 °C to 800 °C (Figure 14). These profiles include samples of Ni-MCM-14, Ni-MCM-48 and Ni-SBA-15 synthesized by incorporation and impregnation, as shown in Figure 15. Additionally, Figure 16 presents the profiles for Ni-MCM-41 with nickel loadings of 3, 6, and 9 wt.% synthesized through incorporation.

The thermal analysis curves provide valuable insights into the two distinct stages of weight loss as the temperature increases. In the range from 30 °C to 100 °C, the weight loss of 1.6% to 2.1% indicates the desorption of physisorbed water on the external surface and the removal of water trapped in the mesopores. In the temperature range of 100 °C to 800 °C, the weight loss, which varies from 1.7% to 5%, is linked to the oxidative decomposition and removal of organic species from surfactants and organo-silica precursors.

A comparative analysis of nickel samples incorporated within molecular sieves was performed using thermogravimetric analysis to evaluate mass losses across two temperature ranges: 30–100 °C and 100–800 °C. In the first range (30–100 °C), mass losses varied from 1.0% to 4.8%. These losses primarily resulted from the desorption of physisorbed water on the external surfaces and the removal of water trapped in the mesopores. Among the samples analyzed, the Ni-MCM-48 exhibited the lowest mass loss, indicating its effective retention of moisture. This phase was characterized by oxidative decomposition and the removal of organic species from precursors, which included surfactants and organosilica. Residual carbon species also contributed to these losses. Additionally, water loss occurred from the condensation of adjacent silanol (Si-OH) groups, leading to the formation of siloxane (Si-O-Si) bonds. In terms of thermal stability, the Ni-MCM-48 sample proved to be the most stable.

The comparison of thermal stability between the synthesized samples produced by incorporation and impregnation methods clearly indicates that the synthesis method has a direct and significant impact on the thermal stability of the Ni-MCM-48 sample. The evidence shows that the Ni-MCM-48 sample synthesized by impregnation possesses lower thermal stability than the sample synthesized by incorporation.

Incorporating a larger amount of metal into the framework of MCM-14 resulted in two distinct stages of weight loss. In the second stage, the mass loss decreased as the metal content increased, from 4.5% to 2.5%. This reduction in mass loss is likely due to the presence of metal, which stabilizes the material’s structure, or the accumulation of Ni species that can hide the silanol species on silica, and thus the Si-OH species are not available for interaction with airborne water molecules.

## 4. Conclusions

MCM-41, MCM-48, and SBA-18 have been successfully synthesized by the sol–gel method. According to the results of this study, the addition of a transition metal such as Ni to molecular sieves’ surfaces affects their textural and morphological characteristics. Using specific characterization techniques, it has been demonstrated that the method of introducing the metal into the mesoporous site is crucial. The incorporation has been more efficient because of the smaller crystallite dimensions and higher intensities of the diffraction peaks. A reduction in the intensity of the peaks and a noticeable broadening of all peaks can be attributed to a decrease in the lattice order caused by the use of the impregnation method. The introduction of metal into the structure of molecular sieves has the effect of decreasing the specific BET surface area, but impregnation has a much greater effect. Depending on the molecular sieve, incorporation results in a decrease in the specific surface of 2.2–20.2%, and impregnation results in a decrease of 14–50%, respectively. According to the SEM investigations, incorporation synthesis results in better metal dispersion than impregnation preparation.

The thermogravimetric studies have revealed that samples that incorporate the metal have higher thermal stability than those synthesized by impregnation, making them more susceptible to adsorption or catalytic reactions. Incorporating a larger amount of metal into the framework of the MCM-14 stabilizes the structure of the sieves.

Manipulating versatile materials, such as mesoporous molecular sieves, during synthesis can result in materials with large specific surface areas or certain pore sizes, which can make them attractive as adsorbents or catalysts in various applications.

## Figures and Tables

**Table 1:** Indexing of the diffraction lines for the molecular sieves.

Sieve	Peak 1			Peak 2			Peak 3			$\bar{LC}$
	2θ	FWHM	LC	2θ	FWHM	LC	2θ	FWHM	LC	
MCM-41	2.64	0.237	35.1	3.1	0.186	45.3	5.15	0.189	45.3	41.9
MCM-48	2.81	0.762	10.9	-	-	-	-	-	-	10.9
SBA-15	0.32	0.367	22.6	1.1	0.186	45.3	-	-	-	34.0

**Table 2:** Indexing of the diffraction lines for the samples synthesized by incorporation and impregnation.

Sample	Peak 1			Peak 2			Peak 3			$\bar{LC}$
	2θ	FWHM	LC	2θ	FWHM	LC	2θ	FWHM	LC	
Ni-MCM-41_inc_	2.55	0.268	30.8	4.47	0.303	27.5	5.20	0.191	43.9	34.1
Ni-MCM-41_imp_	2.70	0.212	39.2	3.15	0.248	33.5	5.3	0.179	46.8	39.8
Ni-MCM-48_inc_	2.21	0.705	11.8	-	-	-	-	-	-	11.8
Ni-MCM-48_imp_	2.80	0.579	14.4	-	-	-	-	-	-	14.4
Ni-SBA-15_inc_	0.33	0.414	20.1	0.95	0.205	40.5	-	-	-	30.3
Ni-SBA-15_imp_	0.31	0.376	22.0	1.08	0.134	60.9	-	-	-	41.5

**Table 3:** Indexing of the diffraction lines for the Ni-MCM-41 synthesized with different metal loadings by incorporation.

Sample	Peak 1			Peak 2			Peak 3			$\bar{LC}$
	2θ	FWHM	LC	2θ	FWHM	LC	2θ	FWHM	LC	
Ni-MCM-41_inc_ (3 wt.% Ni)	2.55	0.268	31.0	4.47	0.303	27.5	5.20	0.191	43.5	34.1
Ni-MCM-41_inc_ (6 wt.% Ni)	2.50	0.289	28.7	4.41	0.306	27.1	5.10	0.201	41.4	32.4
Ni-MCM-41_inc_ (9 wt.% Ni)	2.42	0.317	26.2	4.29	0.327	25.4	4.90	0.270	30.8	27.4

**Table 4:** Pore diameter, pore volume and surface area of molecular sieves.

Sample	d_100_ (nm)	a_0_ (nm)	Dp (nm)	Wt (nm)	Vp (cm^3^g^−1^)	S_BET_ (m^2^/g)
MCM-41	3.34	3.84	2.51	1.3	1.17	1769
MCM-48	3.14	3.61	2.09	1.52	0.78	1592
SBA-15	9.5	10.93	4.74	6.2	0.74	634

Where d_100_ is the d-Spacing of (100) reflection, a_0_ is the unit cell constant, a_0_ = 2d100/√3. Dp is the pore diameter, Wt is the thickness of the pore wall calculated by the difference (a_0_-Dp) and Vp is the pore volume.

**Table 5:** Pore diameter, pore volume, and surface area of samples synthesized by incorporation and impregnation.

Sample	d_100_ (nm)	a_0_ (nm)	Dp (nm)	Wt (nm)	Vp (cm^3^g^−1^)	S_BET_ (m^2^/g)
Ni-MCM-41_inc_	3.23	3.71	2.40	1.31	1.12	1532
Ni-MCM-41_imp_	3.61	4.15	2.00	2.15	0.81	1512
Ni-MCM-48_inc_	3.99	4.59	2.03	2.56	0.65	1271
Ni-MCM-48_imp_	3.15	3.62	1.88	1.74	0.52	792
Ni-SBA-15_inc_	9.53	10.96	4.45	6.51	0.63	620
Ni-SBA-15_imp_	9.08	10.44	3.72	6.72	0.38	418

**Table 6:** Pore diameter, pore volume, and surface area of Ni-MCM-41_inc_ with different metal loadings.

Sample	d_100_ (nm)	a_0_ (nm)	Dp (nm)	Wt (nm)	Vp (cm^3^g^−1^)	S_BET_ (m^2^/g)
Ni-MCM-41 (3 wt.%)	3.23	3.71	2.40	1.31	1.12	1532
Ni-MCM-41 (6 wt.%)	3.25	3.74	2.26	1.48	1.05	1510
Ni-MCM-41 (9 wt.%)	3.38	3.89	2.11	1.78	1.01	1479

## Data Availability

The original contributions presented in this study are included in the article. Further inquiries can be directed to the corresponding author.
